# How Labeling of Commercial Baby Foods Impacts Parents’ Beliefs About Sugar Content and Related Purchasing and Feeding Decisions: Protocol for a Scoping Review

**DOI:** 10.2196/70135

**Published:** 2025-06-19

**Authors:** Rana Conway, Tiffany Denning, Andrew Steptoe, Clare Llewellyn

**Affiliations:** 1 Research Department of Behavioural Science and Health University College London London United Kingdom

**Keywords:** scoping review, sugar, food labels, baby food, commercial infant food, nutrient warning labels, sugar warning labels, weaning

## Abstract

**Background:**

Average sugar consumption among young children in the United Kingdom exceeds the recommended intake. Many parents choose commercial baby foods believing these to be a healthy option. However, surveys show many products contain high levels of added or free sugars, despite labeling suggesting they are “natural” and “healthy.” Analysis of labels and studies with parents suggest changes such as removing misleading marketing or adding sugar warning labels may impact parents’ beliefs and food choices. However, the literature does not provide a comprehensive understanding of the range of changes to commercial baby food labels that might best support parents in choosing healthier foods for their children.

**Objective:**

This scoping review will explore the published and unpublished evidence base to better understand what is known about how labeling of baby foods impacts parents’ beliefs about a product’s sugar content and related purchasing and feeding decisions.

**Methods:**

The JBI guidelines for methodology of scoping reviews will be followed, and results will be reported using PRISMA-ScR (Preferred Reporting Items for Systematic Reviews and Meta-Analyses Extension for Scoping Reviews) guidelines. The population, concept, and context (PCC) framework will be used to determine eligibility criteria. The search will include various research methodologies, including both quantitative (observational and interventional) and qualitative studies. An initial search of MEDLINE (Ovid) and Embase (Ovid) was conducted to develop a full search strategy for MEDLINE, which is presented here. In addition to MEDLINE and Embase, we will search PsycINFO (Ovid), CINAHL (Ebsco), Web of Science (Core Collection) and the Cochrane Library. Reference lists of included studies will also be searched. Unpublished reports will be identified using Google, Google Scholar, relevant websites, policy statements, and government reports and by contacting relevant government and third-sector organizations. In a 2-stage process, 2 reviewers will independently screen titles and abstracts and then full texts. One reviewer will then extract data and a second will verify accuracy. Findings will be presented in tables and diagrams accompanied by a narrative summary.

**Results:**

The literature searches yielded 2071 records from 6 databases, with 1123 documents remaining after deduplication. The gray literature search used a customized Google search, a targeted search of 34 websites, and contact with 49 experts.

**Conclusions:**

We present a protocol for a scoping review to explore the evidence base to understand what is known about how the labeling of baby foods impacts parents’ beliefs about sugar content and related purchasing and feeding decisions. The results of the review will help policymakers better understand regulatory opportunities to improve the labeling of commercial infant foods to help families feed infants and young children lower-sugar diets.

**International Registered Report Identifier (IRRID):**

DERR1-10.2196/70135

## Introduction

Average sugar consumption among young children in the United Kingdom exceeds the recommended intake, contributing to poor dental health and excess weight gain in the early years [1-3]. Free sugars account for around 10% of calories consumed by children aged 1 to 3 years in the United Kingdom—double the recommendation that free sugar intake should not exceed 5% of total energy [3]. Free sugars include all added sugars; all sugars naturally present in fruit juices, purees, and similar products, in which the structure has been broken down; all sugars in drinks (except dairy-based drinks); and lactose and galactose added as ingredients [3].

Many parents trust that commercial baby foods will contain less sugar than other foods, including home-prepared foods [4]. However, products marketed for infants and young children (aged ≤36 months) often contain high levels of added or free sugars [5-7]. It can be difficult for parents to identify these high-sugar products because of the widespread use of “health halo” statements and imagery that suggest products are healthier than their nutrient profile would indicate, for example, “natural,” “organic,” and “well-balanced,” along with pictures of fruit [8,9]. A survey of labeling on 3427 baby foods across 27 European countries found half included the message “no added sugar,” 35% of which contained free sugars [10].

The World Health Organization considers existing regulation of the composition, packaging, and promotion of baby food to be insufficient and has proposed a number of recommendations, including clearer front of pack labeling (FOPL) on products with a high sugar content [11]. The United Kingdom’s Childhood Obesity Strategy also highlights this issue and makes a commitment to ensure the sugar content of foods is communicated more clearly on food labels, while pointing out that leaving the European Union will provide greater flexibility for regulatory changes [12].

Adding a high-sugar indicator (ie, a sugar warning label) to products is one way to help consumers recognize high-sugar products while also incentivizing product reformulation [11]. A systematic review and meta-analysis of 156 studies concluded that mandatory FOPL policies for traffic lights, nutrition scores, nutrition warnings, or health warnings encouraged children and adults to purchase healthier products in place of less healthy products [13]. A separate scoping review, focusing specifically on experimental studies of nutrient warning labels on sugar-sweetened beverages and ultraprocessed foods, found that sugar warning labels helped adults and children to identify high-sugar products and discouraged them from purchasing these products [14]. While these reviews bring together evidence regarding adults and children making food choices for themselves, no reviews could be found on the impact of FOPL policies for sugar on parents choosing foods for their infants and young children. A particular consideration when choosing baby food products is the general trust parents have in baby food companies and consequently their products [4]. The widespread use of “health halo” statements on high-sugar baby foods, (eg, “no added sugar” or “contains only natural sugar”), as well as listing fruit or concentrated fruit juice rather than “sugar” in ingredient lists, may also inform beliefs about the sugar content of products [6,11]. However, it is unclear if changing particular label features would support parents in feeding their children diets with a lower sugar content.

The aim of this scoping review is to explore the published and unpublished evidence base to better understand what is known about how labeling of commercial baby foods impacts parents’ and other primary caregivers’ beliefs about sugar content and their related purchasing or feeding decisions. This will allow policymakers to better understand regulatory opportunities to improve the labeling of commercial baby foods to support families in feeding infants and young children lower-sugar diets.

## Methods

### Design

The methodology for this scoping review will be underpinned by the JBI methodology [15] and reported using the PRISMA-ScR (Preferred Reporting Items for Systematic Reviews and Meta-Analyses Extension for Scoping Reviews) guideline [16]. A preliminary search of MEDLINE, PROSPERO, and the Cochrane Database of Systematic Reviews was conducted, and no current or currently underway systematic reviews or scoping reviews on the topic were identified.

The review questions are as follows:

1. What is known about how primary caregivers understand nutritional labeling used on commercial baby food and beverages to describe sugar?
2. What is known about how primary caregivers might use sugar warning labels on commercial baby foods and beverages?

### Eligibility Criteria

The population, concept, and context (PCC) framework will be used to determine studies eligible for inclusion (Textbox 1) [17].

**Eligibility criteria.**

**Inclusion criteria**

- Population: parents or primary caregivers of children aged between 4 and 36 months. The ages of 4 to 36 months were chosen as some products are labeled as appropriate from 4 months [7].
- Concept: the key concepts addressed in the scoping review are baby food (any commercially prepared food or beverage labeled as suitable for consumption by children aged ≤36 months), sugar (mono- and disaccharides, including glucose, fructose, and sucrose found in fruits), food labeling (any information, symbols, or statements on packaging, including, but not limited to, nutrition panels, ingredient lists, traffic light labels, and warning labels), and understanding (how parents interpret, understand, or use labels, including pictures, nutrient or health-related statements, and any other packaging information that may influence perceptions of a product’s sugar content).
- Context: studies conducted in northern, western, and southern Europe; North America; Australia; and New Zealand to increase the generalizability of findings to the UK population. These criteria are guided by a previous Public Health England review [18].

**Exclusion criteria**

- Population: studies including broader age groups without presenting subgroup analysis for the target age group of 4 to 36 months.
- Concept: studies/reports focused exclusively on commercial milk formula, product sales, or parents’ perceptions of baby food marketing beyond packaging.
- Context: studies conducted in larger geographical regions without presenting subgroup analysis for target areas.

### Types of Evidence

This scoping review will consider both experimental and quasi-experimental study designs, including randomized controlled trials, nonrandomized controlled trials, and before-and-after studies. In addition, we will consider analytical observational studies, including prospective and retrospective cohort studies, case-control studies, and analytical cross-sectional studies, as well as descriptive observational study designs, including case series, individual case reports, and descriptive cross-sectional studies for inclusion.

Qualitative studies will be considered, including, but not limited to, designs such as phenomenology, grounded theory, ethnography, and qualitative descriptive research. In addition to primary research, this review will consider reviews and meta-analyses, opinion papers, non–peer reviewed reports by government departments and agencies, and third sector organizations.

### Search Strategy

The search strategy aims to locate both published and unpublished studies. An initial search of MEDLINE (Ovid) and Embase (Ovid) was conducted using terms associated with key concepts, such as “parents,” “beliefs,” “sugar,” and “baby food labels.” This initial exploration identified articles investigating the impact of baby food labeling on parental perceptions of sugar content. Words found in titles and abstracts, along with the index terms used to describe the articles, were used to develop a full search strategy for MEDLINE (Table 1). This strategy was collaboratively developed with an academic librarian and will be tailored for each database and information source. Additionally, the reference lists of all included sources of evidence will be screened for additional studies.

To ensure a comprehensive mapping of the literature, a broad range of databases will be searched, including MEDLINE, Embase, PsycINFO (Ovid), CINAHL (Ebsco), Web of Science (Core Collection), and the Cochrane Library. To access unpublished or nonacademic reports, Google and Google Scholar searches will be conducted; relevant websites (eg, the Department of Health and Social Care, the Food Foundation, and Action on Sugar), policy statements, and government reports will be reviewed; and relevant organizations, including those whose websites have been searched, will be contacted.

As this is a scoping review, both process and outcome data will be collected. Potential effect modifiers such as age, gender, ethnicity, and socioeconomic status will also be extracted. Only studies published in English will be considered for inclusion due to time and resource constraints.

In line with Peters et al [17], the search strategy may be modified during the process to include any new terms, concepts, or contexts based on search results and ongoing evidence selection.

**Table 1:** Search strategy for MEDLINE (Ovid).

Search	Terms	Records retrieved^a^
1	exp Parents/ or caregivers/ or grandparents/	197,435
2	(parent* or mother* or caregiver* or father* or grandparent* or grandmother* or grandfather* or guardian*).tw,kf	843,311
3	1 or 2	881,170
4	Infant food/	10,285
5	((baby or babies or preschool* or toddler* or infan*) adj3 (food* or snack* or pouch* or jar or jars or meal* or beverag* or drink* or yoghurt* or cereal*)).tw,kf.	5701
6	4 or 5	14,191
7	Beverages/ or Sugars/ or Dietary sugars/ or Sugar-sweetened Beverages/	24,307
8	((Commercial* or packag* or sugar-sweet* or sugar*) adj3 (food* or snack* or pouch* or jar or jars or meal* or beverag* or drink*)).tw,kf.	21,712
9	(NAS^b^ or sugar* or sucrose*).tw,kf.	237,077
10	Infant/ or Child, preschool/ or (baby or babies or preschool or pre-school or toddler* or infan* or child or children).tw,kf.	2,533,448
11	(7 or 8 or 9) and 10	15,060
12	6 or 11	28,231
13	product packaging/ or exp food packaging/ or product labeling/	16,302
14	((food* or sugar* or nutri* or health* or promotion* or traffic light* or TLL* or FOP* or front-of-pack* or product) adj3 (label* or warning* or information* or claim* or sign* or marketing or packag* or symbol* or sticker*)).tw,kf.	196,710
15	13 or 14	206,516
16	Consumer Behaviour/ or Choice Behavior/	35,240
17	(consumer* behavio?r* or choice behavio?r*).tw,kf.	4505
18	(knowledge* or understand* or choice* or influence* or perception* or perceive* or purchase* or opinion* or attitude* or view* or preference*).tw,kf.	5,427,916
19	16 or 17 or 18	5,436,263
20	3 and 12 and 15 and 19	371
21	limit 20 to english language	326

^a^The search was conducted on May 9, 2024.

^b^NAS: no added sugar.

### Source of Evidence Selection

Following the search, all identified citations will be collated and uploaded to Covidence, an evidence synthesis management tool, where duplicates will be removed. Subsequently, a rigorous 2-step screening process will be undertaken. In stage 1, a pair of independent reviewers will assess the titles and abstracts against the predefined inclusion and exclusion criteria. All studies will be evaluated as “include,” “exclude,” or “unclear.” In stage 2, articles identified as “include” or “unclear” will have their full texts retrieved for assessment against the same inclusion and exclusion criteria. Studies that meet all the predefined criteria will be included in the review.

At both stages, each reviewer will conduct the screening blinded to the other, ensuring unbiased selection. To ensure consistency in our approach, a pilot test will be conducted for both stages of the screening process before completing a review of the literature. Any disagreements between reviewers throughout the process will be resolved though discussion or by consulting an additional reviewer.

A summary of the selection process, with reasons for excluding publications at each stage, will be documented and presented in the final review using the PRISMA-ScR flow diagram [16].

### Data Extraction

The data extraction form, adapted from the JBI methodology for scoping review guidelines [15], will include specific details about the participants, concept, context, study methods, and key findings relevant to the review questions:

- Source, year of publication, and reference
- Year of study
- Country and region
- Aims and purpose
- Population and sample size
- Study methods
- Funding sources
- Key findings that relate to parents’ beliefs regarding sugar content in baby foods, related feeding and purchasing decisions, and any additional relevant insights

This form will be independently piloted by 2 reviewers on 5 included studies and reviewed to determine if any adjustments are required before implementing the form across the remaining studies. Following the pilot phase, one reviewer will independently extract the data from all included studies, while a second reviewer will verify the accuracy of the extracted data. Any disagreements between the reviewers will be resolved through discussion or by consulting an additional reviewer.

### Presentation of Results

As recommended by the JBI, the findings will be presented in tables and diagrams accompanied by a narrative summary [15]. Study findings will be grouped according to primary themes that emerge around each of the 2 research questions.

## Results

Funding was confirmed prior to commencing the scoping review. The literature searches were conducted from database inception to May 2024. As of June 2025, the academic literature search had yielded 2071 records from 6 databases, with 1123 documents remaining after duplicates were removed. The gray literature search used a customized Google search, a targeted search of 34 websites, and contact with 49 experts.

## Discussion

To our knowledge, this will be the first scoping review to explore the evidence base to understand what is known about how the labeling of baby foods impacts parents’ beliefs about sugar content and related purchasing and feeding decisions. The search strategy will include a wide range of electronic databases to reduce the risk of missing relevant publications. Sugar intakes exceed recommendations, and commercial infant foods have been identified as making an important contribution to sugar intake [1-3,7]. Additionally, this topic is of interest to public health policy and consumer research; therefore, the search strategy will be supplemented by searching relevant sources in the gray literature. Adhering to the JBI methodology and PRISMA-ScR guidelines will ensure rigor in our review [15].

A limitation of the proposed scoping review is the exclusion of material not published in English, which may limit the generalizability of the results. While we have set out a clear protocol, we recognize that the search strategy or extraction methodology may require modification. Further refinements will be made as necessary during the ongoing data extraction process to better capture content relevant to the scoping review. Any modifications to the protocol will be detailed when the scoping review is reported. A risk of bias assessment is an important element of systematic reviews but will not be undertaken, as the objective of this scoping review is to map the available evidence rather than assess the internal validity of the evidence.

There are increasing calls for policies to curb the rising rates of childhood overweight and obesity [11]. An important element of the package of policy levers currently being discussed is the way food is labeled, as clear and honest labeling is essential to support consumers in making appropriate food choices [7,11,12]. Dietary habits, once established, are difficult to change; therefore, policies focused on improving dietary habits in the early years could be particularly impactful [3]. The results of the proposed scoping review will provide valuable insights into what is already known about the impact of labels on parents’ perceptions of the sugar content of baby food. This will help policymakers to better understand regulatory opportunities to improve the labeling of commercial baby foods to help families feed infants and young children lower-sugar diets. Results will also help focus future research investigating how commercial infant food labels can best support healthy food choices.

## Abbreviations

FOPL: front of pack labeling
PCC: population, concept, and context
PRISMA-ScR: Preferred Reporting Items for Systematic Reviews and Meta-Analyses

## References

[1] Diet and nutrition survey of infants and young children, 2011. Department of Health & Food Standards Agency.

[2] NDNS: time trend and income analyses for years 1 to 9. Department of Health & Food Standards Agency.

[3] Feeding young children aged 1 to 5 years. Scientific Advisory Committee on Nutrition.

[4] Isaacs A, Neve K, Hawkes C (2022). Why do parents use packaged infant foods when starting complementary feeding? Findings from phase one of a longitudinal qualitative study. BMC Public Health.

[5] Hutchinson J, Rippin H, Threapleton D, Jewell J, Kanamäe Haidi, Salupuu K, Caroli M, Antignani A, Pace L, Vassallo C, Lande B, Hildonen C, Rito AI, Santos M, Gabrijelcic Blenkus M, Sarkadi-Nagy Eszter, Erdei G, Cade JE, Breda J (2021). High sugar content of European commercial baby foods and proposed updates to existing recommendations. Matern Child Nutr.

[6] Garcia AL, Menon R, Parrett A (2022). Extensive use of on-pack promotional claims on commercial baby foods in the UK. Arch Dis Child.

[7] (2019). Foods and drinks aimed at infants and young children: evidence and opportunities for action. Public Health England.

[8] Brunacci K, Salmon L, McCann J, Gribble K, Fleming C (2023). The big squeeze: a product content and labelling analysis of ready-to-use complementary infant food pouches in Australia. BMC Public Health.

[9] Gallagher-Squires C, Isaacs A, Reynolds C, Coleman PC (2023). Snacking practices from infancy to adolescence: parental perspectives from longitudinal lived experience research in England. Proc Nutr Soc.

[10] Grammatikaki E, Wollgast J, Caldeira S (2021). High levels of nutrients of concern in baby foods available in Europe that contain sugar-contributing ingredients or are ultra-processed. Nutrients.

[11] Ending inappropriate promotion of commercially available complementary foods for infants and young children between 6 and 36 months in Europe. World Health Organization.

[12] (2016). Childhood obesity: a plan for action. Department of Health and Social Care.

[13] Song J, Brown MK, Tan M, MacGregor GA, Webster J, Campbell NRC, Trieu K, Ni Mhurchu C, Cobb LK, He FJ (2021). Impact of color-coded and warning nutrition labelling schemes: A systematic review and network meta-analysis. PLoS Med.

[14] Taillie LS, Hall MG, Popkin BM, Ng SW, Murukutla N (2020). Experimental studies of front-of-package nutrient warning labels on sugar-sweetened beverages and ultra-processed foods: a scoping review. Nutrients.

[15] Peters M, Godfrey C, McInerney P, Munn Z, Tricco AK (2020). Chapter 11: scoping reviews. JBI Manual for Evidence Synthesis.

[16] Tricco A, Lillie E, Zarin W, O'Brien Kelly K, Colquhoun H, Levac D, Moher David, Peters Micah D J, Horsley Tanya, Weeks Laura, Hempel Susanne, Akl Elie A, Chang Christine, McGowan Jessie, Stewart Lesley, Hartling Lisa, Aldcroft Adrian, Wilson Michael G, Garritty Chantelle, Lewin Simon, Godfrey Christina M, Macdonald Marilyn T, Langlois Etienne V, Soares-Weiser Karla, Moriarty Jo, Clifford Tammy, Tunçalp Özge, Straus Sharon E (2018). PRISMA Extension for Scoping Reviews (PRISMA-ScR): checklist and explanation. Ann Intern Med.

[17] Peters M, Godfrey C, McInerney P, Khalil H, Larsen P, Marnie C, Pollock Danielle, Tricco Andrea C, Munn Zachary (2022). Best practice guidance and reporting items for the development of scoping review protocols. JBI Evid Synth.

[18] (2019). Foods and drinks aimed at infants and young children: evidence and opportunities for action: appendix 2: a rapid scoping review examining the role and impact of commercial baby foods and drinks on the diets of children aged 4-36 months. Public Health England.

